# Impact of robotic-assisted and video-assisted sympathetic nerve reconstruction on quality of life for severe compensatory hyperhidrosis after thoracic sympathectomy

**DOI:** 10.1093/icvts/ivad106

**Published:** 2023-06-14

**Authors:** Dorian Rojas, Simon M Duggan, Marion Mauduit, Amedeo Anselmi, Jean-Philippe Verhoye, Simon Rouze, Jean Valla, Bertrand Richard De Latour

**Affiliations:** Department of Thoracic and Cardiovascular Surgery, Pontchaillou University Hospital, Rennes, France; Department of Thoracic and Cardiovascular Surgery, Pontchaillou University Hospital, Rennes, France; Department of Thoracic and Cardiovascular Surgery, Pontchaillou University Hospital, Rennes, France; Department of Thoracic and Cardiovascular Surgery, Pontchaillou University Hospital, Rennes, France; Department of Thoracic and Cardiovascular Surgery, Pontchaillou University Hospital, Rennes, France; Department of Thoracic and Cardiovascular Surgery, Pontchaillou University Hospital, Rennes, France; Department of Thoracic and Cardiovascular Surgery, Pontchaillou University Hospital, Rennes, France; Department of Thoracic and Cardiovascular Surgery, Pontchaillou University Hospital, Rennes, France

**Keywords:** Sympathetic nerve reconstruction, Compensatory hyperhidrosis, Robotic-assisted surgery

## Abstract

**OBJECTIVES:**

Severe compensatory hyperhidrosis (CH) is a disabling complication following thoracic sympathectomy. Our study was to establish valid patient selection criteria and determine outcomes of nerve reconstructive surgery. Furthermore, we assessed the clinical feasibility and safety of a robotic-assisted approach compared to video-assisted thoracoscopic surgery.

**METHODS:**

Adults with severe CH following bilateral sympathectomy for primary hyperhidrosis were enrolled. We performed 2 questionnaires: the Hyperhidrosis Disease Severity Scale and the Dermatology Life Quality Index before and 6 months after nerve reconstructive surgery. A one-time evaluation of healthy volunteers (controls) was undertaken to validate the quality of life measures.

**RESULTS:**

Fourteen patients (mean age 34.1 ± 11.5 years) underwent sympathetic nerve reconstruction. None of the patients had a recurrence of primary hyperhidrosis. Improvement in quality of life was reported in 50% of patients. Both mean Hyperhidrosis Disease Severity Scale and mean Dermatology Life Quality Index were significantly reduced compared to preoperative assessments. In 10 patients, a video-assisted approach and in 4 patients robotic assistance was utilized. There was no significant difference in outcomes between approaches.

**CONCLUSIONS:**

Somatic–autonomic nerve reconstructive surgery offers a reversal in the debilitating symptoms in some patients with severe CH. Proper patient selection, preoperative counselling and management of expectation are of paramount importance. Robot-assisted thoracic surgery is an alternative method to conventional video-assisted surgery. Our study provides a practical approach and benchmark for future clinical practice and research.

**Figure:**
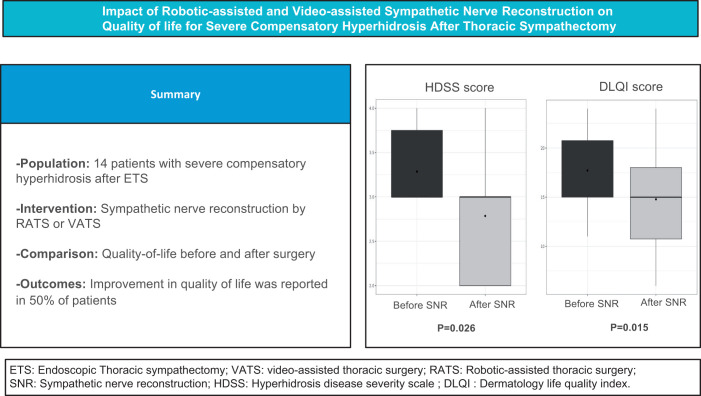

## INTRODUCTION

Compensatory hyperhidrosis (CH) is a common adverse side effect following endoscopic thoracic sympathectomy (ETS) for primary hyperhidrosis [1]. Indeed, in ∼10% of operated patients, they report a severe worsening of symptoms greater than the original complaint with both their quality of life (QOL) and social well-being impaired [2]. This side effect is mainly responsible for patients’ dissatisfaction in the long term and desire for surgical reversal [3, 4].

The mechanism remains unknown but it is thought to result from a dysfunctional reflex arc from the sympathetic nervous system to the hypothalamus, manifesting as excessive sweating in the unaffected areas prior to ETS [5]. Most non-operative treatments for CH are ineffective [6]. There is little consensus on the most appropriate strategy and a lack of standardized outcome measures coupled with the varied results makes management of these patients extremely challenging. One such treatment is sympathetic nerve reconstruction (SNR) with somatic–autonomic nerve grafting to restore the previously transected or ligated neural pathways [7]. The advent of robotic-assisted surgery may potentially improve the outcomes of this procedure, as it facilitates a procedure that closely mirrors the principles advocated in peripheral nerve repair [8].

The purpose of our study was to establish a standardized, practical approach to patients with severe CH in our centre, to determine their outcomes following SNR and, finally, assess the clinical feasibility and safety of a robotic-assisted approach compared to conventional video-assisted thoracic surgery (VATS) treatment.

## MATERIALS AND METHODS

### Ethical statement

The University Hospital Rennes’ Ethics Committee approved the study. All participants provided informed written consent.

### Study population

Inclusion criteria included all adult patients who had undergone bilateral ETS for primary hyperhidrosis and subsequently developed severe compensatory sweating that had failed medical treatment. Patients had undergone ETS at any one of the thoracic centres in France and then were referred to University Hospital Rennes for SNR surgery. Both medical and surgical teams thoroughly reviewed all patients to determine the severity of symptoms and the psychological impact of the disorder and determine the failure of medical management. Patients were counselled and underwent baseline assessment of symptoms with questionnaires prior to the operation and at 6-month follow-up. For the current study, we retrospectively reviewed the prospectively collected data on all consecutive patients between September 2015 and December 2021. The patients enrolled before 2019 were operated by VATS and thereafter by robot-assisted thoracic surgery (RATS). The study adheres to the ‘Strengthening the Reporting of Observational Studies in Epidemiology’ statement guidelines [9, Appendix].

### Controls

To validate the QOL measures, we conducted a prospective study of healthy volunteers to obtain a range of normal sweating values for comparison with patients with CH. We only included individuals aged 18–35 years, with normal body max index, on no medical treatment, who did not smoke and should therefore have normal physiological sweating and a Hyperhidrosis Disease Severity Scale (HDSS) score of one. Controls undertook a single-time evaluation with the HDSS and Dermatology Life Quality Index (DLQI) forms [10].

### Questionnaires

The study protocol used 2 tools to evaluate symptoms and their impact on QOL (Supplementary Material, Appendix).

### Hyperhidrosis disease severity score

The HDSS is a primary hyperhidrosis specific scale that provides a qualitative measure of the effect of patients’ symptoms on their daily activities [11]. The effect of symptoms on daily activities is graded in order of severity from 1 to 4: (1) sweating is never noticeable and never interferes with daily activities; (2) sweating is tolerable and sometimes interferes with daily activities; (3) sweating is barely tolerable and frequently interferes with daily activities; and (4) sweating is intolerable and always interferes with daily activities.

### Dermatology life quality index

The DLQI is a practical, validated questionnaire for assessing QOL in patients with a variety of dermatologic conditions, including primary hyperhidrosis [12]. The DLQI is a 10-question survey that is scored on a scale of 0–30, with higher scores reflecting greater impairment.

### Outcomes

The primary end point was the changes in QOL and symptoms after SNR. Secondary measures included operative outcomes: mortality, morbidity, surgical approach, nerve graft, nerve length, nerve coaptation method, operation length, prolonged chest drain insertion, bleeding and length of stay.

### Statistical analysis

All analyses were performed using R Statistical Software (v4.1.2; R Core Team 2021). Normal distribution was tested using the Shapiro–Wilk test. Continuous data are presented as mean, standard deviation, median and interquartile ranges and were compared using paired and unpaired Student’s *t*-test, or a Wilcoxon rank sum test. Categorical data were expressed as a percentage and compared with Fisher’s test. We used a Wilcoxon signed-rank test to compare HDSS and DLQI results pre- and post-operation. A *P*-value of <0.05 was considered statistically significant.

## SURGICAL PROCEDURE (Video 1)

### Preoperative management

All patients had preoperative assessment of sweating localization and severity. Only patients with severe compensatory sweating were considered for reconstructive surgery. Patients were given both detailed written and verbal information on the procedure and alternative non-surgical options.

### Procedure

**Figure 1:**
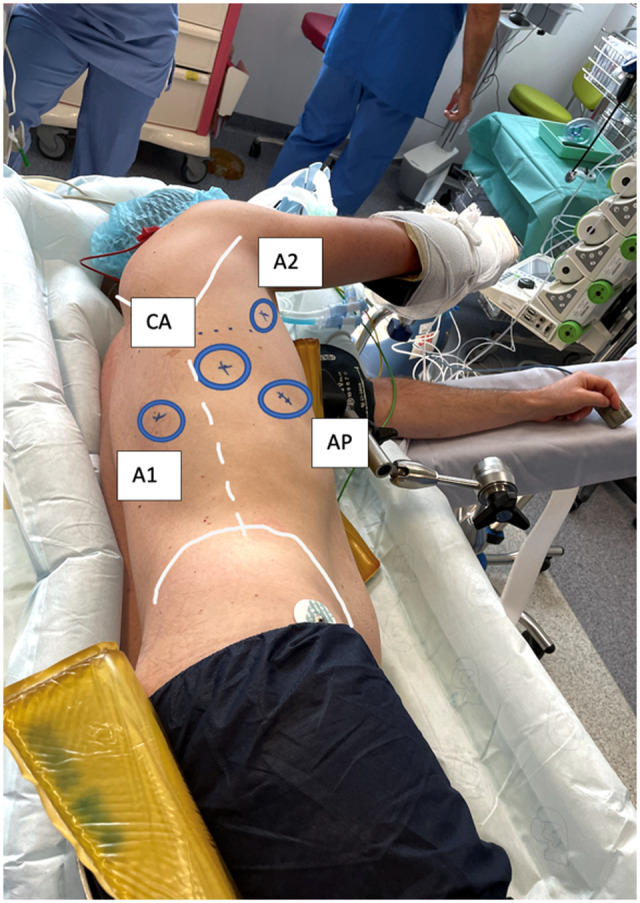
Patient installation and trocar placement. A1 and A2: robotic arm access; AP: assistant port access; CA: camera port access.

Under general anaesthesia with single-lung ventilation, patients were placed in the lateral decubitus position (Figure 1). For the robotic-assisted surgery, the equipment included a DaVinci Xi system (Intuitive Surgical, USA) and a 30° laparoscope. For the VATS approach, we used a 10-mm and 30° laparoscope in addition to a high-definition video thoracoscopic column.

### Step 1: trocar placement

**Figure 2:**
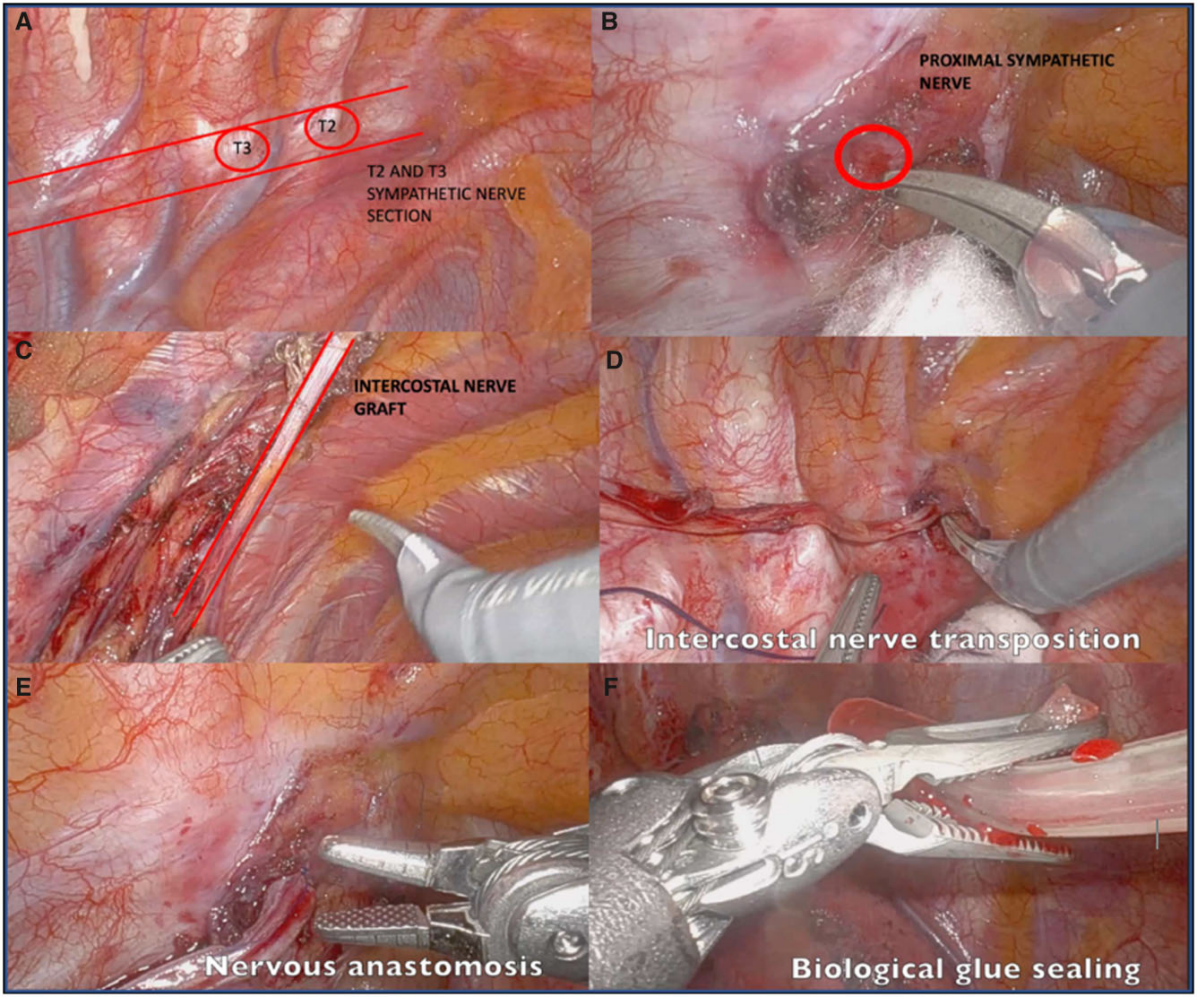
Step-by-step robotic-assisted sympathetic nerve transplantation.

Three 8-mm skin incisions and one 10-mm incision are created (Fig. 2). We place 3 robotic arms: 2 for instruments (A1 and A2) and 1 camera port (CA), in addition to an assistant port (AP) on the 10-mm incision. Following thoracoscope insertion, the sympathetic chain and ganglia are identified at the level of the previous intervention (Fig. 2A). All pneumopleural adhesions are identified and divided.

### Step 2: proximal section of sympathetic nerve dissection

In the initial step, we use a bipolar Maryland and a monopolar hook to identify the ETS proximal section of the sympathetic nerve (Supplementary Material, Appendix, Video 1). When the nerve is fully dissected and neurolysis is completed, it is cleanly cut with robotic scissors. This enables an improved nerve anastomosis (Fig. 2B).

### Step 3: intercostal nerve graft preparation

The intercostal nerve immediately after the distal sympathetic section is identified. The size of the nerve graft is assessed intraoperatively with a silk thread and measured. The intercostal nerve dissection is performed dorsalo-anteriorly with particular caution not to injure the nerve and to preserve the intercostal artery and vein pedicle (Fig. 2C).

### Step 4: transposition of the intercostal nerve

The intercostal nerve is then cut and transposed to the proximal sympathetic cut-section. It is essential that there is no tension on the transposed nerve as this can prohibit nerve regeneration (Fig. 2D).

### Step 5: intercostal nerve anastomosis and biological glue sealing

The intercostal nerve is then anastomosed end to end to the proximal end of the sympathetic nerve with 4 or 5 epineural 8/0 Prolene sutures (Fig. 2E). Biological glue such as CoSeal^®^ Surgical Sealant (Angiotech Biomaterials Corp, USA) is applied to the anastomosis (Fig. 2F). During the VATS procedure, we performed sealing of the nerve with biological glue only. Following haemostasis, a 24-French chest drain is placed posteriorly in the pleural cavity.

### Postoperative management

Postoperative pain management includes an opioid-free multimodal analgesia, a proton pump inhibitor and prophylactic enoxaparin. On the first postoperative day, in the absence of any complication, we aim to remove the chest drain and send the patient home. Surgery for the contralateral side was performed in the same manner for all patients.

## RESULTS

### Patient characteristics

Between September 2015 and December 2021, 14 patients with severe CH underwent SNR at our centre. Preoperative baseline characteristics and details of the preceding ETS are summarized in Table 1. Prior diagnoses included palmar hyperhidrosis in 10 patients with the remaining 4 having had craniofacial hyperhidrosis with erythrophobia. Bilateral sympathectomy had been performed in all patients. All the patients had clinical and psychological examinations with the initiation of cognitive behavioural therapy if necessary prior to consideration for SNR.

**Table 1:** Baseline patient characteristics and sympathectomy data

Patient characteristics and sympathectomy variables	Value (*n* = 14)
Age (years), median (IQR)	31 (27; 34)
Gender, *n* (%)	
Male	10 (71)
Female	4 (29)
BMI, mean ± SD	24 ± 2.80
Age of onset, *n* (%)	
0–10 years	11 (79)
11–20 years	3 (21)
Primary hyperhidrosis distribution, *n* (%)	
Palmar	10 (71)
Craniofacial	4 (29)
Type of surgery for PHH, *n* (%)	
Bilateral sympathectomy	14 (100)
T2 resection only	3 (21)
T2–T3 resection	6 (43)
T2–T4 resection	5 (36)

BMI: body mass index; IQR: interquartile range; PHH: primary hyperhidrosis; SD: standard deviation.

### Operative details and study outcomes

Demographic information and outcomes are listed in Table 2. There were 4 females and 10 male patients with a mean age of 34 ± 11.5 years. The most common sites of compensatory sweating were chest, back, abdomen and thigh. The mean duration between the ETS and nerve reconstructive surgery was 54 ± 13 months. VATS was performed in 10 cases and RATS in 4 cases. The proximal and distal stumps of the transected sympathetic nerve were identifiable in all patients. Reconstruction was performed with the intercostal nerve in 8 patients (57%) and sural nerve in 6 patients (43%). Seven out of the 8 patients (88%) with an intercostal nerve transfer reported an improvement in CH symptoms, whereas none of the patients with the sural nerve reported an improvement. Two out of the 6 patients with a sural nerve graft had temporary lateral leg and foot dysesthesia. In the intercostal nerve transfer patients, there was no postoperative intercostal neuralgia nor chronic chest pain. Table 3 shows the operative details and a comparison of VATS with RATS. The mean operating time was 95.1 ± 15.5 min. There was not a significant difference in operating time between RATS and VATS (104 ± 25 vs 92 ± 9 min; *P* = 0.5). None of the RATS procedures required conversion to VATS nor thoracotomy or the need for further port insertion. There was no operative mortality. The mean nerve graft length was 6.83 ± 2.98 cm. There was no significant difference between mean nerve graft length used in patients undergoing RATS or VATS approaches (respectively, 6.5 ± 0.7 and 7.0 ± 3.8 cm; *P* = 1). Suturing was undertaken in RATS patients and fibrin glue was used in all patients.

**Table 2:** Procedural details and study outcome

Case no.	Sex	Age (years) at reintervention	Area of CH	Interval from ETS to SNR (months)	SNR procedure	Nerve graft	Reduction in CH
1	F	57	Abdomen, back	6	VATS	Sural	No
2	M	24	Chest, back, thigh	23	VATS	Sural	No
3	M	31	Chest, back	21	RATS	Intercostal	Yes
4	M	27	Back, thigh	69	VATS	Intercostal	Yes
5	M	29	Back, abdomen	47	VATS	Sural	No
6	M	34	Back, abdomen	26	VATS	Intercostal	Yes
7	M	26	Chest	90	VATS	Intercostal	Yes
8	M	23	Chest, back	36	VATS	Sural	No
9	M	62	Perineal, thigh	32	RATS	Intercostal	No
10	F	35	Chest	11	VATS	Intercostal	Yes
11	M	35	Chest, back, thigh	178	RATS	Sural	No
12	M	34	Back, chest	72	VATS	Sural	No
13	M	31	Back, abdomen	128	RATS	Intercostal	Yes
14	M	30	Back, abdomen,	17	VATS	Intercostal	Yes

CH: compensatory hyperhidrosis; ETS: endoscopic thoracoscopic sympathectomy; F: female; M: male; RATS: robot-assisted thoracic surgery; SNR: sympathetic nerve reconstruction; VATS: video-assisted thoracic surgery.

**Table 3:** Operative details and comparison of video-assisted thoracic surgery and robot-assisted thoracic surgery sympathetic nerve reconstruction

Variable	All (*n* = 14)	VATS (*n* = 10), mean, SD	RATS (*n* = 4), mean (SD)	VATS versus RATS, *P*-value
Operating time (min)	95.1 ± 15.5	91.5 ± 8.89	104 ± 25.7	0.52
Nerve bridging distance (cm)	6.83 ± 2.98	7.00 ± 3.81	6.5 ± 0.7	1
Nerve coaptation				
Suturing	4	0	4	
Coagulant/fibrin glue	14	10	4	
Complications				
Bleeding requiring reintervention	1	1	0	
Chest tube duration (days)	1.57 ± 0.852	1.70 ± 0.949	1.25 ± 0.500	0.42
Length of stay (days)	2.36 ± 0.842	2.30± 0.949	2.50 ± 0.577	0.20
Impact on DLQI Score	14.8 ± 5.74	14.4 ± 5.95	15.8 ± 5.91	0.62

DLQI: Dermatology Life Quality Index; RATS: robot-assisted thoracic surgery; SD: standard deviation; VATS: video-assisted thoracic surgery.

One patient (case no. 1) developed a postoperative haemothorax requiring reintervention for haemostasis and a delayed discharge. The mean duration of chest drain insertion was 1.57 ± 0.85 days. No patient had a prolonged duration of chest drain. The mean hospital length of stay was 2.36 ± 0.84 days.

### Quality of life scores

Follow-up was complete for all patients. The improvement in sweating after nerve reconstruction was rated as excellent or good in 7 (50%), unchanged in 6 (43%) and worse in 1 patient (7%) at 6 months (Table 2). None of the patients had a recurrence of primary hyperhidrosis symptoms.

The HDSS and DLQI scores reduced significantly after SNR compared with preoperative values (Table 4). Characteristics of the SNR and healthy controls are shown in Table 5. In a comparison between these groups, the HDSS and DLQI scores were significantly higher in CH patients (Table 6). We also found a significant difference favouring intercostal nerve compared to sural nerve use in postoperative DLQI (11.6 ± 4.6 vs 19 ± 4, *P* = 0.02) (Table 7). Finally, we found no correlation between VATS or RATS surgical approach on univariate analyses (Table 8).

**Table 4:** Hyperhidrosis Disease Severity Scale and scores before and after sympathetic nerve reconstruction

	Before SNR (*n*=14)	After SNR (*n*=14)	Δ mean	*n*	*P*-Value
HDSS, mean ± SD	3.29± 0.47	2.79 ± 0.80	−0.500	14	0.026
DLQI, mean± SD	17.5± 3.87	14.8± 5.74	−2.93	14	0.015

DLQI: Dermatology Life Quality Index; HDSS: Hyperhidrosis Disease Severity Scale; SD: standard deviation; SNR: sympathetic nerve reconstruction.

**Table 5:** Baseline patient characteristics between the sympathetic nerve reconstruction group and the control group

Patient characteristics	SNR group (*n* = 14)	Control group (*n* = 14)	*P*-Value
Age (years), median (IQR)	31.0 (27.5; 34.8)	28.5 (24.5; 30.8)	0.13
Gender, *n* (%)			
Male	10 (71)	10 (71)	1
Female	4 (29)	4 (29)	1
BMI, mean ± SD	24.0 ± 2.80	22.9 ± 2.02	0.33
HDSS, mean ± SD	3.29± 0.47	1 ± 0	<0.001
DLQI, mean ± SD	17.7 ± 3.87	0.214± 0.579	<0.001

BMI: body mass index; DLQI: Dermatology Life Quality Index; HDSS: Hyperhidrosis Disease Severity Scale; IQR: interquartile range; SD: standard deviation; SNR: sympathetic nerve reconstruction.

**Table 6:** Validating Hyperhidrosis Disease Severity Scale and scores before sympathetic nerve reconstruction

	Controls (*n*=14)	CH before SNR (*n*=14)	Δ mean	*n*	*P*-Value*
HDSS, mean ± SD	1 ± 0	3.29± 0.47	−2.29	14	<0.001
DLQI, mean ± SD	0.214± 0.579	17.7 ± 3.87	−17.5	14	<0.001

* Versus controls.

DLQI: Dermatology Life Quality Index; HDSS: Hyperhidrosis Disease Severity Scale; SD: standard deviation; SNR: sympathetic nerve reconstruction.

**Table 7:** Impact of intercostal nerve on Dermatology Life Quality Index score

	Mean (SD)	Median (IQR)	Min	Max	*n*	*P*-Value
Intercostal graft						
Yes	11.6 (4.81)	11.5 (7.50; 15.5)	6.00	18.0	8	**0.023**
No	19.0 (4.00)	19.5 (15.8; 21.8)	14.0	24.0	6	–

IQR: interquartile range; SD: standard deviation.

The Bold represents statistically significant.

**Table 8:** Robotic assistance compared to video-assisted thoracic surgery on Dermatology Life Quality Index score

	Mean (SD)	Median (IQR)	Min	Max	*n*	*P*-Value
Robot						
No	14.4 (5.95)	14.5 (10.8; 17.8)	6.00	24.0	10	0.62
Yes	15.8 (5.91)	16.5 (13.2; 19.0)	8.00	22.0	4	–

IQR: interquartile range; SD: standard deviation.

## DISCUSSION

The present study elicited 3 main findings. First, reversal of sympathectomy with an autologous nerve graft improved CH symptoms in 50% of patients. Second, this improvement was observed in those patients who underwent somatic–autonomic nerve grafting with an intercostal nerve. Third, the use of robotic assistance is clinically feasible, although a larger cohort and long-term follow-up, and is needed to delineate positive prognostic factors.

### Symptom and outcome evaluation

Many aspects of the disease presentation and management are affected by patient interpretation and behaviour, from the level of distress of the symptoms to the desire for sympathectomy reversal. External factors affect individuals differently, depending on circumstances rather than physiology, including cultural norms, occupational demands and local climate [2]. We therefore ensured that patients had both a proper trial of conservative treatment and preoperative counselling. Furthermore, we used 2 scoring systems to assess treatment satisfaction. It was important to demonstrate their ease of use, applicability and local validity. To this end, we showed that both the HDSS and DLQI scoring systems can discriminate well between healthy controls and CH patients. Moreover, patients understood them and that they could easily be incorporated into the Thoracic outpatient department consultation. However, these patient-related outcome measures have not been uniformly adopted by all clinical groups perhaps owing to the perceived need for an objective clinical threshold rather than a graduated scale. Further work is needed to define objective physiological end points.

### Nerve reconstruction of the sympathetic chain

Somatic–somatic nerve grafting is a long-established treatment for peripheral nerve injury [13]. Its principles have been applied to the reconstruction of the thoracic sympathetic chain in both experimental and clinical series [8, 14, 15]. However, in contrast, the results of CH treatment have been modest and highly variable. For patients with prior sympathectomy, somatic–autonomic nerve reconstruction has a probability of CH symptom improvement in the order of 10–40% [8]. This is directly comparable with our results, whereby we observed an improvement in both the HDSS and DLQI scores in 50% of our cohort. We also found no pattern of anatomical area or dermatomal distribution that appeared to improve compared to others. The explanation for our findings is likely multifactorial and follows a similar reasoning as when there is limited nerve regeneration following neurotmesis repair [16]. Biological factors such as Wallerian degeneration, neural atrophy and fibrosis, and the effects of chronic axotomy on denervated tissues are known to prohibit complete nerve recovery [16]. If there has been associated soft tissue injury or extensive nerve damage during the primary ETS procedure, this can result in significant scarring and distortion, both of which result in the persistence of conduction block [17] Additional negative prognosticators include: age of patient, prolonged duration between primary ETS procedure and SNR and proximity of nerve transection to distal target. In a future study, a larger sample size might be able to delineate the significance of these factors. Finally, the interval to neural recovery after SNR is not well defined [2]. Several months, may be required to regenerate autonomic neural tissue, ushering delayed success of reversal in some patients that was not identified at follow-up. Some animal models have attested to this with sympathetic nerve regeneration after grafting only becoming evident after 6 months [14, 15].

### Nerve graft

We used 2 types of autologous nerves for the reconstruction of the postganglionic sympathetic neurons. These act as immunogenically inert scaffolds, providing appropriate neurotrophic factors, adhesion molecules and viable Schwann cells for axonal regeneration [18]. The choice of graft is dependent on the size of the nerve gap, location of proposed nerve repair and associated donor-site morbidity [17]. The sural autograft is the most commonly used nerve for peripheral somatic–somatic repair. However, in contrast to intercostal nerve transfer, it requires an additional incision, more extensive dissection, and increased use of electro-cautery. Coupled together, harvesting of the sural nerve is therefore more liable to inadvertent damage and potential chronic sensory deficits. However, we observed only temporary dysesthesia and no chronic complications in our patients. Although our study was not powered to detect a difference between nerve types, we found that in all but 1 of the intercostal nerve transfers the symptoms improved. In contrast, none of the patients who had sural nerve interposition had such an outcome. There are potentially several reasons for this observation. First, the intercostal nerve contains more sympathetic nerve fibres than the sural nerve. [8]. Second, the intercostal nerve is harvested and transposed as a vascularised pedicle. This allows the nerve graft to avoid the initial period of ischaemia and ensures continuous nutrition. Moreover, intraneural fibrosis is avoided and axonal regeneration and target connectivity is enhanced [19]. Third, it is estimated that at each coaptation site, there is 50% loss of axons [16]. Thus, for an intercostal nerve transfer with 1 coaptation site, approximately 50% of the original axons will successfully regenerate through the anastomosis. In contrast, for a sural nerve graft with 2 coaptation sites, only 25% of axons will successfully regenerate. Finally, regardless of the chosen nerve, poor regeneration may occur at the repair site due to unavoidable size and fascicle mismatch, scarring and fibrosis from sutures and tissue handling [17].

### Robotic and thoracoscopic approaches

There are no studies directly comparing SNR outcomes between the different surgical approaches. The earlier reports looked at nerve reconstruction using a video thoracoscopic approach, autologous intercostal nerve transposition and a fibrin glue anastomosis [20, 21]. However, in these retrospective studies, there was excessive loss to follow-up and the use of unvalidated patient-reported outcome measures made it difficult to draw any meaningful conclusions. Moreover, in about 10% of cases, technical factors such as extensive adhesions or inadequate interposition graft length or multiple ganglia transection were causes of failed reconstruction. There have been other case reports of nerve graft with fibrin glue anastomosis or the use of autologous superficial arm vein as the conduit with thoracoscopic interposition with mixed results [22, 23].

Recent studies have looked at outcomes using robotic assistance [24, 25]. Through high-definition visualization, 10× magnification and articulating micro instrumentation within the thorax, the robot is asserted to enable easier identification of the sympathetic trunk, division of adhesions and neurolysis and permit epineural suture anastomosis. The advantage of microsuture repairs is the ability to perform a precise coaptation whilst preserving tensile strength [17]. Against this however, the suture material may itself cause tissue reactions, including inflammation and scar formation, which reduce nerve regeneration [26]. Evidence to support sutureless coaptation with tissue adhesives was recently provided by a meta-analysis showing that nerve regeneration was similar between the 2 techniques [27]. Perhaps combining fibrin glue with 1 or 2 positional sutures might allow for a precise realignment of the nerve fibres and provide sufficient tensile strength to avoid dehiscence, whilst minimizing fibrosis.

In our study, both RATS and VATS showed the same outcomes, albeit in small patient numbers. There were no significant differences between secondary end points of operative time, length of stay or complication rates. As this was our preliminary experience, the number of RATS procedures is low but compares with other published work [25, 26]. However, our follow-up was longer and with more in-depth QOL assessments. Further work is needed to demonstrate the potential benefits of RATS specific to SNR and whether there exists a benefit of anastomosis over sutureless coaptation with tissue adhesives.

### Limitations

Our study has several limitations that could lead to confounding. First, is a monocentric study, it can lead to selection bias. The findings, therefore, may not be extrapolated to other centres and further validation is needed. Second, as the only surgical for SNR in France, we are reliant upon the patient information from the referral centres, which could lead to information bias. Third, the sample size is small and heterogenous. Therefore, strong conclusions cannot be made. Furthermore, only stipulations about non-inferiority between thoracoscopic and robotic techniques can be made. Fourth, we compared outcomes only at 6 months postoperatively, which may have not been sufficient to observe meaningful nerve regeneration and regaining of function. The present findings should be validated in a larger and longer prospective matched cohort study.

## CONCLUSION

In conclusion, we present a detailed description of SNR in the treatment of CH. In particular, we have shown the clinical feasibility of using micro-suturing techniques using robotic assistance in its treatment. Furthermore, we have validated the assessment tools and outlined a practical approach to patients with CH. This should provide a benchmark for further clinical practice and research.

## Supplementary Material

ivad106_Supplementary_Data

## Data Availability

The data underlying this article will be shared on reasonable request basis.

## Notes

Presented at the 2022 EACTS Meeting, Milan, Italy, 6 October 2022.

## ABBREVIATIONS

CH: Compensatory hyperhidrosis
DLQI: Dermatology Life Quality Index
HDSS: Hyperhidrosis Disease Severity Scale
QOL: Quality of life
ETS: Endoscopic thoracic sympathectomy
RATS: Robot-assisted thoracic surgery
SNR: Sympathetic nerve reconstruction
VATS: Video-assisted thoracic surgery
