# Supplementary material for: Impact of robotic-assisted and video-assisted sympathetic nerve reconstruction on quality of life for severe compensatory hyperhidrosis after thoracic sympathectomy
Source: Interdiscip Cardiovasc Thorac Surg. 2023 Jun 14;36(6):ivad106. doi: 10.1093/icvts/ivad106 (PMC10371036; doi:10.1093/icvts/ivad106)
Supplement: ivad106_Supplementary_Data [file ivad106_Supplementary_Data.zip › HDSS.pdf]

INTERNATIONAL HYPERHIDROSIS SOCIETY®
Taking action. Taking care.

www.SweatHelp.org

Hyperhidrosis Disease Severity Scale

"How would you rate the severity of your hyperhidrosis?"

❑ 1.	My sweating is never noticeable and never interferes with my daily activities
❑ 2.	My sweating is tolerable but sometimes interferes with my daily activities
❑ 3.	My sweating is barely tolerable and frequently interferes with my daily activities
❑ 4.	My sweating is intolerable and always interferes with my daily activities

Tips and Usage Information for Clinicians

The Hyperhidrosis Disease Severity Scale (HDSS) is a disease-specific, quick, and easily-understood diagnostic tool that provides a qualitative measure of the severity of the patient's condition based on how it affects daily activities. The questions that make up the scale may be posed to the patient in written or interview form.

Ask the patient to select the statement above that best reflects his or her experience with sweating of the specified body area. Next to each statement is a number. These numbers indicate how responses should be scored. A score of 3 or 4 indicates severe hyperhidrosis. A score of 1 or 2 indicates mild or moderate hyperhidrosis. Post-treatment, as part of a measurement of treatment efficacy and patient satisfaction, the HDSS may be administered again. A 1-point improvement in HDSS score has been associated with a 50% reduction in sweat production and a 2-point improvement with an 80% reduction.

The validity and reliability of the HDSS have been analyzed using three studies and have been found to have strong to moderate correlations with the Hyperhidrosis Impact Questionnaire (HHIQ), Dermatology Quality of Life Index (DLQI), and gravimetric sweat production measurements.

Reference: A Comprehensive Approach to the Recognition, Diagnosis, and Severity-Based Treatment of Focal Hyperhidrosis: Recommendations of the Canadian Hyperhidrosis Advisory Committee, Dermatologic Surgery, August 2007, pages 908-923
