# Supplementary material for: Impact of robotic-assisted and video-assisted sympathetic nerve reconstruction on quality of life for severe compensatory hyperhidrosis after thoracic sympathectomy
Source: Interdiscip Cardiovasc Thorac Surg. 2023 Jun 14;36(6):ivad106. doi: 10.1093/icvts/ivad106 (PMC10371036; doi:10.1093/icvts/ivad106)
Supplement: ivad106_Supplementary_Data [file ivad106_Supplementary_Data.zip › dermatology-life-quality-index-dlqi-2.pdf]

DERMATOLOGY LIFE QUALITY INDEX (DLQI)

Hospital No: é é é é é é é é é é é é é é . **Date:** é é é é é é é é .

Name: é é é é é é é é é é é é é é . **Score:** é é é é é é é é .

Address: é é é é é é é é é é é é é é . **Diagnosis:** é é é é é é é é .

é é é é é é é é é é é é é é .

The aim of this questionnaire is to measure how much your skin problem has affected your life OVER THE LAST WEEK. Please tick (✓) one box for each question.

1.	Over the last week, how **itchy**, **sore**, **painful** or **stinging** has your skin been?	Very much	❐		
		A lot	❐		
		A little	❐		
		Not at all	❐		
2.	Over the last week, how **embarrassed** or **self conscious** have you been because of your skin?	Very much	❐		
		A lot	❐		
		A little	❐		
		Not at all	❐		
3.	Over the last week, how much has your skin interfered with you going **shopping** or looking after your **home** or **garden**?	Very much	❐		
		A lot	❐		
		A little	❐		
		Not at all	❐	Not relevant	❐
4.	Over the last week, how much has your skin influenced the **clothes** you wear?	Very much	❐		
		A lot	❐		
		A little	❐		
		Not at all	❐	Not relevant	❐
5.	Over the last week, how much has your skin affected any **social** or **leisure** activities?	Very much	❐		
		A lot	❐		
		A little	❐		
		Not at all	❐	Not relevant	❐
6.	Over the last week, how much has your skin made it difficult for you to do any **sport**?	Very much	❐		
		A lot	❐		
		A little	❐		
		Not at all	❐	Not relevant	❐
7.	Over the last week, has your skin prevented you from **working** or **studying**?	Yes	❐		
		No	❐	Not relevant	❐
	If "No", over the last week how much has your skin been a problem at **work** or **studying**?	A lot	❐		
		A little	❐		
		Not at all	❐		
8.	Over the last week, how much has your skin created problems with your **partner** or any of your **close friends** or **relatives**?	Very much	❐		
		A lot	❐		
		A little	❐		
		Not at all	❐	Not relevant	❐
9.	Over the last week, how much has your skin caused any **sexual difficulties**?	Very much	❐		
		A lot	❐		
		A little	❐		
		Not at all	❐	Not relevant	❐
10.	Over the last week, how much of a problem has the **treatment** for your skin been, for example by making your home messy, or by taking up time?	Very much	❐		
		A lot	❐		
		A little	❐		
		Not at all	❐	Not relevant	❐

Please check you have answered EVERY question. Thank you.

©AY Finlay, GK Khan, April 1992 www.dermatology.org.uk, this must not be copied without the permission of the authors.

DERMATOLOGY LIFE QUALITY INDEX (DLQI) - INSTRUCTIONS FOR USE

The Dermatology Life Quality Index questionnaire is designed for use in adults, i.e. patients over the age of 16. It is self explanatory and can be simply handed to the patient who is asked to fill it in without the need for detailed explanation. It is usually completed in one or two minutes.

SCORING

The scoring of each question is as follows:

Very much	scored 3
A lot	scored 2
A little	scored 1
Not at all	scored 0
Not relevant	scored 0
Question 7, óprevented work or studyingô	scored 3

The DLQI is calculated by summing the score of each question resulting in a maximum of 30 and a minimum of 0. The higher the score, the more quality of life is impaired.

HOW TO INTERPRET MEANING OF DLQI SCORES

0 ï 1	no effect at all on patient's life
2 ï 5	small effect on patient's life
6 ï 10	moderate effect on patient's life
11 ï 20	very large effect on patient's life
21 ï 30	extremely large effect on patient's life

REFERENCES

Finlay AY and Khan GK. Dermatology Life Quality Index (DLQI): a simple practical measure for routine clinical use. *Clin Exp Dermatol* 1994; **19:**210-216.

Basra MK, Fenech R, Gatt RM, Salek MS and Finlay AY. The Dermatology Life Quality Index 1994-2007: a comprehensive review of validation data and clinical results. *Br J Dermatol* 2008; **159:**997-1035.

Hongbo Y, Thomas CL, Harrison MA, Salek MS and Finlay AY. Translating the science of quality of life into practice: What do dermatology life quality index scores mean? *J Invest Dermatol* 2005; **125:**659-64.

There is more information about the DLQI, including over 85 translations, at www.dermatology.org.uk. The DLQI is copyright but may be used without seeking permission by clinicians for routine clinical purposes. For other purposes, please contact the copyright owners.

©AY Finlay, GK Khan, April 1992 www.dermatology.org.uk, this must not be copied without the permission of the authors.
